# Adapting the Social Norms Exploration Tool in the Democratic Republic of the Congo to Identify Social Norms for Behavior Change

**DOI:** 10.9745/GHSP-D-24-00058

**Published:** 2024-10-29

**Authors:** Kathryn Sugg, Florence Mpata, Radha Rajan, Dominick Shattuck, Dédé Aliango Marachto, Peter J. Winch

**Affiliations:** a Johns Hopkins School of Public Health Center for Communication Programs/Breakthrough ACTION, Baltimore, MD, USA.; b Le Centre de Recherche Interdisciplinaire en Développement: Economie, Santé, et Société-Université Protestante au Congo, Kinshasa, Democratic Republic of the Congo.; c Johns Hopkins Bloomberg School of Public Health, Baltimore, MD, USA.

## Abstract

In the Democratic Republic of the Congo (DRC), male engagement, social norms, and social networks mitigate family planning behavior. We discuss the adaptation of the Social Norms Exploration Tool (SNET), which identifies relevant social norms and community members upholding these norms, to inform the development of family planning interventions in the DRC. The SNET provides activity tools and templates to guide users through the following steps: (1) plan and prepare, (2) identify reference groups, (3) explore social norms, (4) analyze results, and (5) apply findings.

The SNET approach resulted in discussion of social norms, particularly around birth spacing and gender norms framing the man as the decision-maker. However, despite applying a methodology specifically designed to identify social norms, other factors limiting use of contraceptive methods were identified in the process, including lack of education, rumors, and misconceptions. Adaptations were needed to include the full range of reference groups due to narrow phrasing of primary questions, and some of the participatory methods were overly complicated. Feedback from experienced data collectors suggested that the social norms framework is not intuitive, is difficult to apply correctly, and may require that data collectors have a stronger foundation in the relevant concepts to produce valid and actionable results.

Although the SNET provides language for discussing normative factors and techniques to identify reference groups and social norms, modifications to the implementation process are recommended when adapting the tool for research.

## Abstract (precis)

The Social Norms Exploration Tool is a rapid assessment tool for programs that research can adapt to help determine how to explore social norms and identify which norms and population interventions to address to facilitate the behavior of interest.

## Teaser

**Key Findings:**

- The Social Norms Exploration Tool (SNET) can be modified to render the findings more useful and more actionable, as well as to make the tool more straightforward to implement for future users.
- Our experience will help other programs and researchers adapt the tool for more generalizable qualitative research and recommendations.
- While the SNET has been used for program teams, it is also useful for thinking through full-protocol formative research on social norms.

**Key Implications:**

- The SNET provides a structure for identifying reference groups, but this article provides guidance to ensure all important populations are captured and highlights the different roles that different reference groups may play.
- Project implementers or donors doing formative research on social norms can learn from the practical, on-the-ground experience of applying the SNET.

## INTRODUCTION

### Social Norms and Family Planning in the Democratic Republic of the Congo

Social norms are unwritten rules of behavior shared by members of a group or community. They dictate what people believe is normal (descriptive norms) and appropriate or approved (injunctive norms) behavior.^1^ Power hierarchies that favor men over women are manifest in restrictive social and gender norms that negatively influence women’s health and well-being.^2^ Research, globally and in the Democratic Republic of the Congo (DRC) specifically, indicates limited spousal communication about health and pervasive norms that position men as decision-makers, inhibiting women’s access to health services.^3^^,^^4^ A secondary analysis of the 2013–2014 Demographic Health Survey in the DRC found that 54.1% of women surveyed said their partner alone was the decision-maker for the respondent’s health care,^5^ and that women who had no say in their health care were less likely to be users of modern contraception than women who made their own health care decisions.^6^

Men’s lack of communication around family planning contributes to social norms that discourage adoption of family planning. These social norms include perceptions that family and community members do not support family planning and that there is pressure to have more children. Systematic reviews of qualitative research on family planning and social norms in sub-Saharan Africa identify social support and gender norms as influencing factors. A review of 13 qualitative studies on contraceptive use among young people found that social support for contraception was a motivator for contraceptive uptake, while social norms against discussing contraception acted as societally based barriers.^7^ In a review of 23 qualitative studies of determinants of condom use among adolescents, 13 studies noted that gender norms affected the practice. Two studies in the review highlighted that negative peer norms discourage condom use among young men.^8^

The Social Norms Exploration Tool (SNET; https://www.irh.org/social-norms-exploration) was developed by the Institute for Reproductive Health at Georgetown University as a rapid assessment tool to aid program implementers in conducting qualitative, participatory activities to better understand the social norms that influence behavior in a community and that are of interest to their project. It includes 5 steps to explore social norms and inform interventions: (1) plan and prepare, (2) identify reference groups, (3) explore social norms, (4) analyze results, and (5) apply findings. Tools and templates, which are intended to be modified, are provided for each step.

The Social Norms Exploration Tool is a rapid assessment tool to aid program implementers in better understanding the social norms that influence behavior in a community.

The SNET was validated via pilot testing with 2 projects (Masculinité, Famille, et Foi and “Growing up GREAT!”) in Kinshasa, DRC, from 2015 to 2019, before being tested in other countries.^9^ From 2018 to 2020, the SNET was applied by the Passages Project in Burundi, Niger, and the DRC,^10^^–^^12^ by Save the Children in Nigeria, Burkina Faso, and Palestine,^13^^–^^15^ by the MOMENTUM project in Nigeria,^16^^,^^17^ and by the U.S. Agency for International Development (USAID)-funded Tulonge Afya project in Tanzania.^18^ Of the SNET applications for which reports are available, most focused on gender norms around reproductive health, early marriage, or intimate partner violence. In these applications, the SNET was adapted for use in different ways. For example, to identify reference groups, most applications used the SNET’s “My Network Tool,” but other projects have used in-depth interviews, voting during focus group discussions (FGDs), and FGD notes. To explore social norms, several applications used vignettes as the participatory activity, often complemented by the “5 Whys” activity, whereas 2 applications implemented the problem tree analysis only.

This study adapted the SNET and sought to identify the social norms and reference groups influencing male engagement in family planning discussions, decision-making, and uptake of methods to inform programmatic interventions in the DRC. A secondary objective was to further validate the SNET by applying the tool in smaller cities and rural areas in the DRC. We describe the value added by the SNET and challenges to adapting the tool, as well as recommendations for future use as the basis for human subjects qualitative research.

## METHODS

### Adaptation of the Social Norms Exploration Tool

The USAID-funded project Breakthrough ACTION DRC adapted the SNET for this qualitative research study. The study was implemented in 3 DRC provinces with low contraceptive prevalence representing varying regions, ethnic, and linguistic groups: Lualaba, Sankuru, and Kasaï Central.

Applying the SNET in the DRC involved the selection and adaptation of data collection exercises presented in the SNET and the development of data collection instruments.

### Planning and Protocol Development

Following the SNET’s plan and prepare guidance for step 1, we conducted the problem tree analysis with project staff to analyze barriers to contraceptive use among married couples in the DRC and identify root causes of low adoption of modern contraceptive methods. This process, combined with the 5 Whys activity, narrowed down the behaviors and populations of interest and shaped the research questions. The plan and prepare guidance, along with steps 2 and 3 of the guide, informed the development of the study protocol and adaptation of data collection instruments (Table 1).

**TABLE 1.** Adaptation of the SNET Toolkit for Application in the Democratic Republic of the Congo

**SNET Component**	**Process of Adaptation**
Step 1: Planning and preparing	Two SNET activities were selected to determine root causes of low contraceptive prevalence: Problem tree analysis: Staff wrote a selected behavior of interest as the tree trunk and enumerated reasons why people did or did not perform the behavior as the roots and the consequences of performing or not performing the behavior as the branches. Five Whys activity: Team members paired up to pose a “why” question related to the behavior of interest. They asked each other “why” again for every answer for 5 iterations of “why.”
Data collection instrument for Phase 1: Identifying reference groups	SNET activity adapted: “My Social Networks” Central question: “When discussing family planning methods - whether it is questions about what they are, where to get them, how to use them, or whether you or your partner should use them - with whom do you feel comfortable discussing these topics?” The respondent was read a list of options to probe specific attributes of the person chosen. For example, if he said a family member, the questionnaire would skip to questions about how the person was related, if they were a spouse, whether they were older or younger than the participant, and on which side they were related (maternal or paternal). Adaptation: Unlike the example in the SNET, we asked only 1 question, and the participant was only able to share 1 influential person. Recruitment: systematic household sampling rather than convenience sampling. Implementation: A tablet-based digitized questionnaire to quickly analyze responses by site rather than the manual analysis template.
Data collection instruments for Phases 2 and 3: Exploring social norms	The decision of activities to include from the SNET for the qualitative data collection was informed by input from IRH staff. SNET activities selected: 5 Whys activity and vignettes. “Five Whys” activity: Questions were based on barriers identified through the problem tree analysis, and the activity was conducted in small groups with a facilitator rather than in pairs without a facilitator. Participants in focus groups were divided into subgroups and asked 2 initial questions. The second question that all focus group participants were asked was the same: “When a couple has decided to use family planning, some men do not allow their wife or partner to choose freely or independently which family planning method to adopt. Why is this?” Vignettes were developed and shared with the in-country team to be validated for cultural appropriateness and contextual accuracy. During the individual interviews, a vignette was read in full. It described a couple deciding whether to use a family planning method for birth spacing. A shortened version of the vignette was used with the focus groups.

Abbreviations: IRH, Institute for Reproductive Health; SNET, Social Norms Exploration Tool.

### Data Collection

In each province, the sample included individuals from 1 health subdistrict within each of 2 urban and 2 rural health zones. Data were collected in French, Swahili, or Tshiluba by 3 teams of 4 data collectors from the local firm ALMA Research Services, and data collection took place in 3 phases between March 11 and April 1, 2022 (Table 2). Data collectors were assigned to province teams based on their comfort with the region and fluency in the local language. All data collectors had prior experience conducting qualitative research. All data collectors participated in a 5-day training with in-depth review of the study instruments, practice with all instruments and activities, including role-playing, and a module on social norms.

**TABLE 2.** Data Collection Methods Used in Social Norms Exploration Tool Adaptation, 2022, Democratic Republic of the Congo

**Phase**	**Data Collection Method**	**Objective**	**Population**	**No.**
1	Rapid, tablet-based questionnaire	Identify reference groups who influence men around family planning	Married and unmarried men aged 15–39 years and 18–39 years, respectively	317
2	Semistructured, individual interviews	Examine how social norms affect male involvement in family planning Examine men’s experiences with family planning, their involvement in the family planning process, and their perception(s) of the male role in this process	Married and unmarried men aged 15–39 years and 18–39 years, respectively	24
3	Focus group discussions with identified reference groups	Understand how reference groups communicate their attitudes, beliefs, and behaviors about family planning to the men they influence	Friends (of population of interest): men aged 15–39 years Wives (of population of interest): women aged 15–39 years Nurses Doctors Religious leaders	144

The first phase of data collection corresponded to the SNET step 2, identify reference groups. Adapted from the SNET “My Social Networks” activity, the survey was digitized to a tablet-based questionnaire to improve data quality and facilitate rapid analysis of the results (as opposed to tabulating by hand). Unlike the example in the SNET, participants were asked only 1 question and were only able to share 1 influential person with whom they felt comfortable discussing family planning methods. In addition, rather than the SNET approach of selecting fewer participants through convenience sampling, we selected a larger sample through systematic household-based sampling (Table 1). Men (n=317) identified the people in the community with whom they were most comfortable discussing family planning (Table 2).

The second phase of data collection mapped to the SNET step 3, explore social norms. Although the participatory activities in the SNET are designed to be facilitated with groups, the vignettes can be implemented individually, as we did in this adaptation. Semistructured, individual interviews were conducted with a subsample of married and unmarried men (n=24) who had participated in the rapid questionnaires (Table 2). Interview participants were asked open-ended questions about their experiences with and involvement in family planning and their perception of men’s role in this process. In the interviews, a vignette describing a couple contemplating using family planning was used to examine how men in their community would respond to specific situations around birth spacing, couple communication, and permitting their partner to adopt a modern family planning method.

The third phase, a continuation of exploring social norms (step 3), took place several days later. Data collectors analyzed the questionnaire results from the first phase and recruited participants matching the reference group criteria to focus groups. The focus group discussions (FGDs) were designed to understand how reference groups reinforce descriptive and injunctive norms around family planning. A total of 144 participants participated in 24 FGDs, which included the SNET’s 5 Whys activity and a shortened version of the vignette (Table 2). In the DRC adaptation, we chose to facilitate the 5 Whys activity in small groups rather than in pairs, with 1 facilitator assigned to each group. This decision reflected the complexity of the activity in simulations during the data collector training.

### Data Analysis

We adapted the analysis phase from SNET step 4 to accommodate a more conventional qualitative data analysis. While the SNET provided note-taking templates, we supplemented note-taking with audio recordings of qualitative data collection activities, subsequently transcribed into French, to preserve data not captured in the notes. Given the length and number of transcripts, we inductively coded a subset of the transcripts with qualitative analysis software ATLAS.ti and grouped the codes into 6 overarching themes.

Analyses of FGD and interview transcripts occurred through a participatory 5-day workshop that convened data collectors, program staff, and Ministry of Health representatives. The 6 themes were shared with the workshop participants for validation and the addition of any other reoccurring themes based on their reading of the transcripts. Specifically, the participatory analysis workshop included the following steps. Each participant was asked to read 2 transcripts and to discuss their findings with another participant who had been assigned transcripts from the same province and geography (urban/rural). Then, teams of 4 were assigned to read transcripts from each province. The teams came together to discuss similarities and differences between the urban and rural areas in their assigned province and engaged in the development of subthemes (with supporting citations). Finally, each team presented their findings and recommendations for their assigned province in plenary, and the presentations, notes, and supporting citations were shared with the research team.

### Ethical Approval

The study was approved by the Institutional Review Boards of the Johns Hopkins Bloomberg School of Public Health (IRB approval #18667) and the University of Kinshasa School of Public Health (IRB approval # ESP/CE/04/2022). Data collectors were trained on human subjects research. All participants gave their informed consent.

## RESULTS OF THE SOCIAL NORMS EXPLORATION TOOL ADAPTATION

### Assessment of Adapting the Social Norms Exploration Tool for Research

We found it useful to develop a set of criteria with which to evaluate our experience adapting the SNET methods for social norms research. The criteria for analyzing the utility of the SNET-recommended methods make reference to the stated aims of the 5 steps:

1. How did the SNET guidance and activities facilitate development of the research questions, protocol, and instruments?
2. How completely were reference groups identified during phase 1 of data collection?
3. How successful was the adoption and adaptation of recommended participatory activities for exploring social norms during interviews and FGDs?
4. How did SNET results inform development of the data analysis workshop?
5. How effectively did results from the social norms exploration translate into recommendations?

### Phase 1: Identification of Reference Groups

In phase 1, a rapid questionnaire was applied to identify reference groups. Given that the results of phase 1 were the basis for recruiting focus group participants in phase 3, we wanted to avoid the bias that could result from talking to a small convenience sample of men and extrapolating their nominated reference groups to the entire community. Thus, 317 men across the 3 provinces were recruited door-to-door for participation in the questionnaire. Seventy-five percent of the men were married, and all men were aged 15–39 years (Table 3). Results from these questionnaires informed the selection of reference groups in each province.

**TABLE 3.** Demographic Characteristics of Men Recruited to Identify Reference Groups, Democratic Republic of the Congo

	**No. (%)** **(N=317)**
Age, years	
15–19	32 (10.1)
20–24	64 (20.2)
25–29	62 (19.6)
30–34	73 (23.0)
35+	86 (27.1)
Marital status	
Married	239 (75.4)
Not married	78 (24.6)
Province	
Kasaï Central	105 (33.1)
Lualaba	101 (31.9)
Sankuru	111 (35.0)

We asked men to identify with whom they felt most comfortable discussing family planning. For each province and geographic site (urban/rural), we selected reference groups based on the 2 most-cited responses. Table 4 shows the most-cited group, health care providers (47.0%), followed by friends (24.0%) as the second most-cited reference group, and family members (20.2%).

**TABLE 4.** Reference Groups Identified by Province and Health Zone, Democratic Republic of the Congo

	**Reference Group Cited, No. (%)**					**Total, No.**
	**Family Member**	**Friend**	**Community or Religious Leader**	**Health Care Provider**	**Other/Do Not Know**	
Kasaï Central	26 (24.8)^a^	14 (13.3)	8 (7.6)	55 (52.4)^b^	2 (1.9)	105
Bilomba	7 (26.9)^a^	0 (0)	1 (3.9)	18 (69.2)^b^	0 (0)	26
Kananga	6 (21.4)^a^	3 (10.7)	4 (14.3)	14 (50.0)^b^	1 (3.6)	28
Mikalayi	8 (30.8)^a^	5 (19.2)	2 (7.7)	11 (42.3)^b^	0 (0)	26
Ndesha	5 (20.0)	6 (24.0)^a^	1 (4.0)	12 (48.0)^b^	1 (4.0)	25
Lualaba	26 (25.7)	38 (37.6)^b^	4 (4)	27 (26.7)^a^	6 (6)	101
Dilala	8 (32.0)^a^	12 (48.0)^b^	0 (0)	5 (20.0)	0 (0)	25
Fungurume	8 (32.0)^a^	9 (36.0)^b^	2 (8.0)	5 (20.0)	1 (4.0)	25
Kanzenze	6 (24.0)^a^	8 (32.0)^b^	1 (4.0)	7 (28.0)	3 (12.0)	25
Manika	4 (15.4)	9 (34.6)^a^	1 (3.9)	10 (38.5)^b^	2 (7.7)	26
Sankuru	12 (10.8)	24 (21.6)^a^	7 (6.3)	67 (60.4)^b^	1 (1)	111
Bena Dibele	6 (21.4)	7 (25.0)^a^	0 (0)	15 (53.6)^b^	0 (0)	28
Dikungu	0 (0)	8 (29.6)^a^	4 (14.8)	15 (55.6)^b^	0 (0)	27
Lodja	3 (11.1)	8 (29.6)^a^	1 (3.7)	14 (51.9)^b^	1 (3.7)	27
Omendjadi	3 (10.3)^a^	1 (3.5)	2 (6.9)	23 (79.3)^b^	0 (0)	29
**Total**	**64 (20.2)**	**76 (24.0)**	**19 (6.0)**	**149 (47.0)**	**9 (2.8)**	**317**

a Second most cited reason.

b Most cited reason.

Men who were surveyed cited health care providers as those with whom they felt most comfortable discussing family planning, followed by friends and then family members.

### Phases 2 and 3: Exploration of Social Norms

Phases 2 and 3 of the study explored social norms through participatory activities. In Sankuru, religious leaders were not among the most cited groups but were selected for participation due to the emerging theme in the interviews around the importance of religion. Themes from the qualitative data collection activities represented normative factors as well as individual factors, such as knowledge and attitudes (Table 5).

**TABLE 5.** Normative and Other Factors Affecting Family Planning: Themes and Exemplary Quotes

**Theme**	**Illustrative Quotes**
Norms around birth spacing	*It’s always good to have children who don’t follow each other, that we don’t conceive while the other is still little. Because if you can conceive early for a child, it can cause you to suffer a lot; when he will be always sick, today, sick tomorrow…you will only spend money.* —Man, Kasaï Central
Knowledge of family planning methods	*To avoid the woman conceiving while the child is still too young, you can abstain from lying with your wife, or you buy condoms; if you don’t want to buy condoms, you can bring the wife there to the hospital, get an [intrauterine device].* —Man, Kasaï Central
Benefits of birth spacing methods	*Since the woman has seen that her child is 6 months, instead of having the pregnancies close together and causing her child to suffer, she prefers to use a method so that her child will grow up well.* —Man, Kasaï Central
Concerns about modern family planning methods	*My friend came to show me the facts of his wife, who they gave the injection, and that hurt, it really had a bad effect, a lot of hemorrhaging, hemorrhaging that lasted a month.* —Man, Kasaï Central] *He must know the methods favoring the woman to engage in prostitution, that is to say that for the man when the woman takes a method, for him the woman will no longer be afraid of engaging in prostitution.* —Man, Sankuru *They refuse because the woman can choose a method that lasts many days, many years. They want it to be fewer years.* —Woman, Lualaba
Gender norms	*If she goes alone to the health center to get the [contraceptive method], there it’s as if she is a free woman or as if she is the authority in her household…it’s as if her husband has become the wife and she the wife has become the husband.* —Man, Kasaï Central
Reference groups	*There are 2 types of friends. There’s a friend and a close friend. He could have spoken with his close friends, those who he says, night or day, I must talk to them. Those friends, he can tell, sometimes they say, “Don’t do that” and he really listens to their advice.* —Man, Kasaï Central
Sources of information	*Because the woman understands, we give advice, she retains it; or her friends can tell her, or her grandparents; it’s that that she retains and comes to speak to the man, to see how to avoid early pregnancy while the children are still young.* —Man, Kasaï Central
Sanctions for those who act contrary to social norms	*The man can think of taking another wife. Since he sees that his wife has become difficult, he can think about taking another wife.* —Nurse, Sankuru

The results highlighted both descriptive and injunctive norms related to birth spacing, uptake of modern methods, and couples’ communication and decision-making. Birth spacing was a common and approved practice, albeit frequently practiced through traditional methods rather than modern methods. Gender norms invoking men as heads of household gave men authority over their wives and the final say in decisions about birth spacing and family planning method use. Injunctive norms stipulated that men who ceded decision-making power to their wives or whose wives used family planning were seen as weak or impotent. Health care providers who prescribed contraception without the husband’s consent could also face consequences, including the husband bringing a complaint against them.

At the individual level, respondents recognized the health benefits of birth spacing for mothers and children, as well as the economic benefits. Many men expressed concerns about allowing their wives to adopt a modern method or to select the method of her choice. They cited fears around women’s infidelity, negative side effects, and infertility.

Finally, findings reflected the importance of reference groups on male engagement in family planning and the distinction between reference groups and sources of knowledge. Overall, men were most comfortable talking to health care providers about family planning. Close friends served as both sources of knowledge and reference points for descriptive norms. Religious leaders were not cited as often, but religious mores that frame family planning as sinful had clear influence on injunctive norms.

### Phase 4: Analysis

This study expanded beyond the SNET guidance provided for the analysis step to ensure that all the data were captured, rather than relying on note-takers, which could have resulted in inconsistencies across study activities. Because the data collectors did not have a background in social norms before their training for this study, we wanted to ensure that the note-takers did not miss or gloss over potential social norms for lack of recognizing them as norms. During the participatory workshop, participants read the transcripts and discussed emerging themes and insights, first by site, then by province, and finally in plenary.

### Phase 5: Recommendations

Results and recommendations from the participatory workshop were shared through dissemination events in the study provinces. We proposed recommendations to apply the study findings to the development of interventions. We hosted workshops with local health officials to translate findings into program activities that could be added to operational action plans. Deviating from SNET Step 5 (application of findings), we followed a multistep framework to bridge the gap from study findings to evidence-based program planning. Results in the form of themes and exemplary quotations, as well as recommendations by reference group, were shared via dissemination events that were followed by a data use workshop intended to adapt programmatic messaging and generate province-level health communication activities. We incorporated questions from SNET Step 5 into the workshops to help health officials address social norms with their workplan activities.

## DISCUSSION OF THE UTILITY OF THE SOCIAL NORMS EXPLORATION TOOL AS ADAPTED FOR RESEARCH

Overall, the SNET proved to be a useful framework for structuring our study. Taking the time to quantitatively identify with whom men felt comfortable discussing family planning allowed us to rapidly tailor our qualitative data collection to the most important reference groups in each study site. Talking to a range of people across different reference groups provided context and triangulation for the social norms that were raised through interviews with the population of interest. However, other aspects of the SNET were less useful. We discuss lessons learned during the present adaptation of the tool, organized by the SNET step. While the SNET is aimed at program implementers rather than researchers, we feel our experience will be helpful to both groups.

Overall, the SNET proved to be a useful framework for structuring our study.

### Step 1: Plan and Prepare

The SNET provides options for exploring social norms, with limited guidance on how to select the methods. This application of the SNET was among the first to be conducted as a formal research study undergoing ethical review. The SNET does not provide guidance on processes for ethical review of research protocols or sampling appropriate for a qualitative research study conducted across different sites and provinces. While the SNET states that implementers should “hold a team discussion on ethical considerations of the social norms exploration,” it does not discuss obtaining approval from ethical review boards nor anonymity of data. The Breakthrough ACTION DRC project approached the research systematically, in part, due to the need to submit a full protocol to the Institutional Review Board and local ethics committee according to USAID policy.^19^ Utarini et al. list 11 “critical criteria” for evaluating rapid assessment procedures, of which 1 is around ethics and the need to obtain approval from ethical review boards. The lack of explicit recommendations for ethical review and approval in the SNET is a weakness, albeit one that is common across similar rapid assessments.^20^

Based on our experience, we recommend the SNET for conceptualizing social norms research, with specific recommendations for adaptation. For example, the team conducted the problem tree analysis (Table 6) during the planning period. This brainstorming methodology gave surface-level responses rather than root causes behind non-use of family planning methods. Instead, we recommend asking project staff to communicate their experiences or observations of the behavior of interest through narrative storytelling and then elicit root causes from those examples. Similarly, the 5 Whys activity (Table 6) did not result in a deeper understanding of root causes.

**TABLE 6.** Strengths and Weaknesses of Each Methodologic Feature of the SNET

**Method**	**Research Questions**	**Advantages of This Method**	**Threats to Validity**	**Key Insight or Recommendation**
Problem tree analysis	What are the root causes that prevent couples from using modern contraception?	Sparked a conversation about causes of a behavior of interest.	The project team, based in Kinshasa, must try and represent the perspectives of all regions (many far from Kinshasa) and subgroups (a wide range of religious, economic, and ethnic backgrounds).	The project team had to repeatedly reference the SNET to discuss whether causes discussed did or did not focus on norms but felt they had limited resources in the SNET to make those determinations. The root causes often surfaced common sayings (e.g., “children are wealth”); even among project staff, it was difficult to produce answers to why these sayings are widely believed and repeated. It may be easier to derive root causes from examples, experiences, or observations than to enumerate them directly.
Identifiction of reference groups	Were the reference groups identified consistent with the definition of reference groups in the social norms literature?	Rapid analysis of which community members men consider influential to their behavior around family planning. Sample frame is tailored to each site.	Groups identified were not limited to reference groups but included sources of family planning information such as health care providers. Influential groups with whom men do not feel comfortable talking about family planning were not captured	Men are influenced not only by social norms but also by knowledge of family planning methods. To keep the study focused on norms, inclusion criteria for what constitutes a reference group should be developed. Questions should attempt to identify enforcers of social sanctions as well as supporters of the behavior of interest.
Vignette of a couple making decisions about family planning	Did scenarios serve to identify ways in which reference groups influence men’s involvement in and decisions about family planning?	Present a specific scenario for men to respond to rather than an abstract concept. Men may feel more comfortable responding to questions about someone else rather than talking about their own experiences.	Certain aspects of the scenario were confusing or not well understood. Too many hypotheticals made the scenario overly complicated. Some men may not feel comfortable responding to certain questions.	Vignettes were well received overall, during both interviews and FGDs. The version used in the interview guide had too many plot points. Future applications of the SNET should keep the vignette short and straightforward and could use a “fill-in-the-blank” framing to allow participants to co-create the scenario with the facilitators.
Focus groups with site-specific reference groups	Did focus groups effectively identify ways in which reference groups influence men’s involvement in and decisions about family planning?	Triangulation between men’s perceptions of what others in the community expect of them and what these influential persons actually expect. Different reference groups may hold different norms.	Groups identified were not limited to reference groups but included sources of family planning information, such as health care providers. Influential groups with whom men do not feel comfortable talking about family planning were not captured in Phase 1, although the decision to include them was made by the team during data collection.	Health care providers, such as doctors and nurses, while not necessarily reference groups in the way, are defined in the SNET and are still privy and subject to the social norms operating in their communities. Findings from the FGDs provide a fuller picture of the context around family planning norms and practices.
Five Whys exercise	Did exercise identify root causes of why men do or do not engage with family planning decision-making or allow their partners to choose a method independently?	The goal of the exercise was to delve a level further with each “why?” to deepen understanding of why a norm or behavior exists.	In many cases, the 5 Whys provided were 5 separate responses to the initial “why” instead of digging into the underlying norms.	This exercise was too complicated, requiring the moderator and note-takers to decide in the moment which reasons were most in line with a normative framework and pursue that line of questioning. We would not recommend this method in future applications of the SNET.
Participatory analysis workshop	How well did participants understand concepts like social norms, injunctive norms, and sanctions? Did participants benefit from introduction of these concepts? Was the workshop productive in identifying interventions?	Collaboration between different stakeholders. Intercoder discussion at various levels to compare findings from urban and rural sites. Validation of themes developed through inductive coding before workshop.	Participants did not have a deep understanding of social norms, and certain concepts (e.g., the idea of social sanctions) were misunderstood. Social norms are not intuitive, and participants did not find social factors to be the most important influence on behavior. Insufficient time was given for the analysis.	Hosting a participatory analysis workshop may increase buy-in from stakeholders and decision-makers. However, if participants do not see social norms as a priority, it will be difficult to maintain a focus on social factors throughout. Workshops should be longer than 5 days or based only on a subset of data or data that has been distilled or pre-coded.

Abbreviations: FGD, focus group discussion; SNET, Social Norms Exploration Tool.

### Step 2: Identify Reference Groups

The reference group questionnaire allowed for rapid analysis of reference groups and precise tailoring of reference group criteria by site. One challenge is the assumption that those with whom men are most comfortable discussing family planning are those from whom men receive cues about social norms. In the study, men also received cues about social norms from other sources with whom they were less comfortable, such as religious leaders who may reinforce injunctive norms about family planning as sinful. SNET implementers should attempt to take other normative influencers into account.

The reference group questionnaire allowed for rapid analysis of reference groups and precise tailoring of reference group criteria by site.

The SNET and associated guidance stress the importance of identifying specific attributes of reference group members. If a man were to nominate a cousin, follow-up questions would identify the cousin as male or female, maternal or paternal, older or younger. This level of detail was relevant to SNET applications in which data collectors would recruit the specific person cited, as in Nigeria.^13^ However, it did not serve to define categories of reference groups (i.e., older, maternal, male cousins). From a practical standpoint, such a specific reference group is not functionally different from “male peers aged 18–49 years.” Rather than building the questionnaire around 1 question and asking follow-up questions about the specific person cited, future applications should include questions to ascertain who is influential in supporting the behavior of interest, as well as which community members are not supportive. Dichotomizing the reference groups into “enforcers” of sanctions versus “social supporters,” as in Burundi,^10^ would help researchers better understand the social pressures on the population of interest.

### Step 3: Explore Social Norms

FGDs with reference group members were important for understanding the social context, exploring different perspectives, and triangulating findings between groups. Qualitative data from married women, religious leaders, and health providers created a fuller picture of the pressures and norms surrounding family planning access. FGDs identified how reference groups influence men’s involvement in family planning and decision-making.

Vignettes (Table 6) were incorporated into the individual interview and FGD guides. The vignettes represented scenarios that participants recognized from their communities and provided a way for respondents to discuss sensitive issues without speaking to personal experience. However, providing too many plot points was confusing and made it difficult for respondents to understand how each series of questions differed from the series before. The SNET recommends that reference group members be introduced in the vignette. In Nigeria, vignettes included fill-in-the-blanks to allow participants to direct the story in the way they felt was most plausible (“She goes to speak to _____”). This example provides a good option for future applications to capture the role reference groups play.

The second SNET-recommended activity applied only in FGDs was the 5 Whys activity (Table 6), characterized as an activity of easy-to-medium difficulty. While easy to develop, it was confusing to implement. Rather than asking a question and delving further into root causes with each additional “why,” participants gave different, surface-level responses to the initial question. Participants were confused about how each “why” was distinct from the previous iteration. The time spent on the 5 Whys exercise did not add value to the findings, and we do not recommend it to others interested in using the SNET.

### Step 4: Analyze Results

In the “Analyze Results” section of the SNET, data are compiled based on activity templates and notes taken during data collection. Our challenge was moving from raw transcripts to a stage where the data had been sufficiently distilled such that it was possible for the team to apply the SNET guidance for analysis. Given the number of transcripts and the desire for shared ownership of the results, we arranged for collaboration between different stakeholders and intercoder discussion to compare findings across sites. A subset of qualitative transcripts was inductively coded before the workshop, and validation of existing themes and addition of new themes occurred at the end of each day of the workshop. A collaborative synthesis was conducted by small groups corresponding to each province and shared with the rest of the participants at the end of the workshop.

The concept of social norms was not intuitive for the workshop participants. Many participants did not have a deep understanding of social norms, and a brief training was insufficient to prepare the participants to fill out the SNET example templates to rank the relative influence of normative factors. Certain concepts were misunderstood, such as social sanctions as defined in the social norms literature versus negative consequences more generally (for example, experiencing side effects after adopting a family planning method). Despite the SNET focusing on social norms, the participants did not find that social norms were the most important influence on behavior. Many recommendations from the workshop focused on increasing knowledge rather than addressing normative factors. Future applications of the SNET should include activities to better acquaint workshop participants with social norms and provide sufficient time to synthesize norms from the data. One of the key findings reflected in other articles on rapid assessment procedures is the need for capacity-building in social sciences to go hand-in-hand with provision of ethnographic or qualitative research manuals,^21^ with 1 article noting that “general capacity building in the social sciences is necessary to assure the validity and appropriate use of program-specific manuals.”^22^ Spending more time during the data collector training to ensure that all data collectors had a good understanding of social norms and how they operate and doing a short refresher before the data analysis workshop would have better equipped data collectors to focus on identifying social norms rather than other behavioral influences. The unspoken nature of social norms and greater familiarity with other behavioral drivers, such as knowledge and attitudes, presented a challenge to identifying different norms and their function in the community.

### Step 5: Apply Results and Formulate Recommendations

The concluding section of the SNET presents questions for the program team to (1) adjust project design and (2) adjust research, monitoring, and evaluation. The questions provided in the SNET for adjusting project design helped inform the microplanning work by asking participants to identify obstacles based on the findings and identify how to include influential groups in future interventions. Questions, such as “What positive norms can we build on?” “What harmful norms should we address?” and “Which people and (reference) group are influential?,” helped to structure the conversation about adapting the findings from the social norms exploration into activities that could be added to future workplans. Members of the provincial health departments expressed that they appreciated that the research team returned to their provinces to share the results and think about how to apply them, although the SNET guidance says to do this process as a program team rather than with local health officials.

## CONCLUSION AND RECOMMENDATIONS

The SNET was designed as a program planning tool and provides a good starting point for social norms research, but adaptation to research required reworking some steps. The intensive engagement with human subjects necessitates ethical clearance. Project teams may not be familiar with determining sample sizes and submitting to ethics committees. However, the activities in the SNET fall under the category of human subjects research and require implementers to think through ethical processes and set sample sizes during the planning phase.

We recommend the SNET as a framework to help projects develop research questions and instruments for exploring social norms, recognizing that it is meant for programmatic purposes and requires modification and interpretation to serve as the basis for qualitative research. The rapid analysis of reference groups and inclusion of reference group perspectives strengthens the quality of the exploration and provides context as well as triangulation of different norms and social sanctions across groups.

We recommend the SNET as a framework to help projects develop research questions and instruments for exploring social norms, recognizing that it is meant for programmatic purposes.

The experience in DRC offers insights and recommendations for future applications. The SNET is intended to be widely usable for project teams with a range of social norms expertise, stating, “it is not necessary that you have technical expertise in social norms or participatory approaches.” However, the SNET does not provide enough information or support for teams that may have less experience. Regardless of experience with social norms, we feel that it would help to supplement or replace the problem tree analysis with personal reflections and narrative experiences from project staff around the behavior(s) of interest—either based on their own experiences or in their programmatic work. We suggest applying precise language to ask about differing roles played by reference groups rather than only identifying those to whom the respondent would talk about the behavior. For example, asking, “With whom do you feel comfortable talking about family planning? If you were to use family planning with a partner, who would you tell? Who would approve? Who in your community do you think would disapprove?” Responses to these questions can be used to create different sets of reference groups based on whether they are perceived as “enforcers” of sanctions against the behavior or “supporters” of the behavior.

We also recommend that future project implementers or researchers keep vignettes short and easy to follow, using the vignette to set the scene and probing respondents to describe what behaviors and actions would follow. For example, the interviewer could ask which other community members might be involved and how. Incorporating straightforward questions about what characters would do next or who they would involve in their decision-making may provide more information than presenting the complete scenario and asking more complicated questions about motivations or community expectations. We found that conducting FGDs with reference group members as described in the SNET was important for obtaining different perspectives on social norms in the community and suggest that future implementers also include those who may not interact as closely with the population of interest but who nevertheless have an impact on social norms in the community, such as religious or traditional leaders. We recommend omitting the 5 Whys activity.

Finally, we advise integrating activities that increase knowledge of social norms for data collectors and workshop participants ahead of any data analysis workshop, as well as allowing enough time for the participatory analysis workshop such that participants are able to spend time with and discuss the data. While the Breakthrough ACTION DRC application used full transcripts, working from notes and activity templates would have been more manageable.

It is our intent that this in-depth analysis of our own experience adapting the SNET for research purposes will serve as a reference point for other researchers considering future social norms explorations.

## Notes

**Peer Reviewed**

**First Published Online:** October 22, 2024.
